# Ret Finger Protein: An E3 Ubiquitin Ligase Juxtaposed to the XY Body in Meiosis

**DOI:** 10.1155/2009/524858

**Published:** 2010-01-18

**Authors:** Isabelle Gillot, Cédric Matthews, Daniel Puel, Frédérique Vidal, Pascal Lopez

**Affiliations:** ^1^Université de Nice Sophia Antipolis, UFR Sciences, 06002 Nice, France; ^2^INSERM, U636, 06108 Nice, France; ^3^Centre National de la Recherche Scientifique, UMR 6543, PRISM, 06108 Nice, France

## Abstract

During prophase I of male meiosis, the sex chromosomes form a compact structure called XY body that associates with the nuclear membrane of pachytene spermatocytes. Ret Finger Protein is a transcriptional repressor, able to interact with both nuclear matrix-associated proteins and double-stranded DNA. We report the precise and unique localization of Ret Finger Protein in pachytene spermatocytes, in which Ret Finger Protein takes place of lamin B1, between the XY body and the inner nuclear membrane. This localization of Ret Finger Protein does not seem to be associated with O-glycosylation or sumoylation. In addition, we demonstrate that Ret Finger Protein contains an E3 ubiquitin ligase activity. These observations lead to an attractive hypothesis in which Ret Finger Protein would be involved in the positioning and the attachment of XY body to the nuclear lamina of pachytene spermatocytes.

## 1. Introduction

During meiosis, homologous recombination between chromosomes is allowed by synapsis of chromosome's pairs during pachytene stage. At this stage, all the chromosomes are attached to the nuclear membrane by their telomeres. In order to deal with asynchronous synapsis of the XY chromosomes, mammals have adopted a strategy that involves chromatin condensation and seclusion of the sex chromosomes into a specialized and visibly distinct nuclear territory, known as the XY body (see [1, 2] for review). After early pachytene, the XY chromosomal pair becomes highly condensed, transcriptionally silent, and positioned adjacent to the nuclear envelope. 

Localization of the condensed XY body to one part of the nucleus suggests that a particular composition of the nuclear matrix could be involved in the positioning and/or attachment of the XY body or sex chromosomes to the nuclear lamina [3, 4]. The nuclear lamina provides an attachment site for chromatin and facilitates processes associated with DNA replication and transcription. In germ cells, lamin B1, as well as specific isoforms of lamin A/C, is evidenced in the nuclear matrix [5, 6]. 

The precise molecular events underlying sex chromatin condensation and meiotic inactivation of sex chromosomes remain to be elucidated. However some hypotheses have been advanced based on the presence or the absence of specific proteins inside the XY body [2]. Matsuura et al. [7] reported that SUMO-I and PIAS-y are found inside the XY body and proposed that Ret Finger Protein (RFP) can also be associated with the XY body and could then have a role in the silencing of sex chromosomes. 

The initial interest in RFP arose from the demonstration that it becomes oncogenic when fused with the RET tyrosine kinase [8]. In healthy animals, RFP is highly expressed in male germ cells, in particular in primary spermatocytes and in round spermatids [9]. RFP is usually detected as a 58 kDa protein by western blot analysis. However, two isoforms of RFP, 58 kDa and 68 kDa, can readily be detected in testis [9, 10]. Tezel et al. [11] have shown that the 68 kDa isoform results from an O-glycosylation of RFP. Despite extensive studies, the biological functions of RFP remain unclear. It has been described as a nonspecific DNA-binding protein strongly associated with the nuclear matrix [10]. RFP has also been shown to partially colocalize with PML and Inf6 [12, 13]. RFP has been implicated in general transcriptional repression mechanisms, in particular when it interacts with the Enhancer of Polycomb (EPC), Mi-2*β* and histone deacetylase 1 (HDAC1) [14–16]. In somatic cells, SUMO-I modification of RFP induces its relocalization into nuclear bodies reminiscent of the PML bodies and strengthens its transcriptional repressor activity [7]. RFP has also been proposed to repress transcriptional activity of specific proteins such as Retinoblastoma protein or bHLH transcription factors [17, 18]. However, the role of RFP in meiotic cells remains unclear. Finally, RFP belongs to the family of RING-B-Box-Coiled Coil-B30.2 proteins [19]. According to the International Mouse Genome Informatics Committee, the proteins containing RING-B-Box-Coiled Coil motif have been renamed TRIM proteins, and the name of TRIM 27 was assigned to RFP [20, 21]. As a result of numerous studies, it has been proposed that all the RING finger containing proteins, as well as TRIM proteins, could act as E3 ubiquitin ligases and be involved in proteasomal degradation process (reviewed in [20, 22, 23]). 

We report here the precise localization of RFP in pachytene spermatocytes. We demonstrate that RFP exhibits a unique and specific localization that may define the positioning of the XY body. Moreover, as predicted by the presence of a RING domain, we demonstrate that RFP possesses an E3 ubiquitin ligase activity. 

## 2. Materials and Methods

### 2.1. Cells and Virus Propagation

15P-1 cells [24] were maintained at 32°C in DMEM media (Gibco: 41966-029) supplemented with 10% Fetal Bovine Serum (FBS, Gibco: 10270-098) and 1/100 Penicillin/Streptomycin (P-S, Gibco: 15140-163). 

Sf9 cells (BD Biosciences: 554762) were maintained in TNM-FH media supplemented with 1/100 P-S as previously described [25]. Baculoviruses were propagated, prepared, and tittered in Sf9 cells as described by the manufacturer (BD Biosciences). 

Fractionation of germ cells from twenty B6D2F1 mice by elutriation centrifugation was performed as previously described [26] and the purity of each fraction was determined by DNA staining with DAPI. Fractions used were estimated to contain more than 80% of purified spermatocytes, round spermatids, or elongated spermatids.

### 2.2. Construction of Plasmids and Baculoviruses

Primers RFPsense139 5′ CCGGAATTCATGCCTGCGGCCCAGCCG and RFPrev569 5′ CCGCTCGAGGCTGTGGTCGCGGTGCTC were used to amplify the RING-B-Box domain of RFP from total testis cDNA. *Eco*RI/*Xho*I digested PCR fragment was ligated in pGex4T-1 (Amersham Biosciences: 27-4580-01). Transformation of BL21 bacteria (Novagen: 69449) with the pGEX-RING-B-Box resulting plasmid was used for the production of a GST-RING/RFP fusion protein. 

MTS1-Myc baculovirus transfer vector, already described [27], is derived from pAcSG2 (BD PharMingen: 554769) and contains the human cytomegalovirus promoter/enhancer sequences (CMV) and an additional Myc tag. 

RFPsense146StuI 5′ GAAGGCCTCTCATGCCTGCGGCCCAG and RFPrev1733KpnI 5′CGGGGTACCGTCTACGTTCACTTCCTCAC were used to amplify full-length RFP coding sequence. *Stu*I/*Kpn*I PCR fragments were ligated in MTS1-Myc resulting in MTS-Myc-RFP. 

All constructions were verified by sequencing (ABI PRISM 310 genetic analyzer, PerkinElmer Life Sciences). 

Baculoviruses Bac-Myc-RFP encoding for Myc-RFP were generated by cotransfecting Sf9 cells with linearized baculovirus DNA (BD BaculoGold^TM^ transfection Kit: 554740) and MTS-Myc-RFP baculovirus transfer vector as described by the manufacturer. In 15P-1 cells infected with the Bac-Myc-RFP recombinant baculoviruses, CMV promoter drives the expression of Myc-RFP.

### 2.3. Protein Purification in Bacterial Cell System

BL21 bacterial cells transformed with pGEX vectors were amplified at 37°C in LB medium supplemented with 0.1 *μ*M Zn^2+^ and 50 *μ*g/mL ampicillin. At optical density 0.6–0.8, cells were induced at 30°C by adding 1 mM IPTG for two hours. GST fusion proteins were purified using Glutathione Sepharose 4B (Amersham Biosciences: 17-0756-01), eluted with reduced glutathione (Sigma: G-4251), and dialyzed against PBS (Phosphate-Buffered Saline pH 7.4) containing 1 *μ*M ZnSo_4_ using disposable dialyzer (Sigma-Aldrich: Z368385-10EA).

### 2.4. TPEN Treatment of GST-RING/RFP

For cation chelation, GST-RING/RFP immobilized on Glutathione Sepharose was incubated overnight at 4°C, with 5 mM N,N,N′,N′-tetrakis(2-pyridylmethyl)-ethylenediamine (TPEN) (Sigma-Aldrich: 87641) or 1% ethanol (vehicle) in 50 mM Tris, pH 7.5. After extensive washes, one half of the immobilized GST-RING/RFP was treated with 30 *μ*M of ZnCl_2_ for 45 minutes at room temperature. After washing, samples were used for ubiquitination assays.

### 2.5. RFP Immunoprecipitation from Baculovirus-Infected Cells

15P-1 cells, seeded 15 to 18 hours before infection at 80%–90% confluency, were exposed in TNM-FH media to 1 plaque-forming units per cell of wild type or Bac-Myc-RFP recombinant baculoviruses. After 2 hours, the inoculum was replaced by fresh DMEM medium containing 5 mM sodium butyrate (Sigma-Aldrich: B5887) and cells were incubated at 32°C for 72 hours. Cells were lysed in a buffer containing 50 mM Tris-HCl (pH 8.0), 150 mM NaCl, 1% NP-40, and EDTA-free “complete” protease inhibitors (Roche: 1873580) sonicated briefly, and centrifuged for 10 minutes at 13,000 rpm at 4°C (Biofuge pico, 3325 Heraeus rotor; Sorvall, Norwalk, Conn.). Supernatant was used for RFP immunoprecipitation using a rabbit polyclonal antibody to RFP (IBL Japan: JP 18791) according to the supplier's instructions. After washing, immunoprecipitated RFP was used for ubiquitination assays.

### 2.6. Western Blots

Elutriated germ cells [26] were lysed. Protein concentration was evaluated using a Bradford assay (Bio-Rad: 500-0006); 20 *μ*g of proteins per lane were subjected to SDS Page electrophoresis (8%) and blotted on PVDF membrane. Rabbit polyclonal antibody to RFP (IBL Japan: JP 18791) and mouse anti-rabbit peroxidase-conjugated antibody (Sigma-Aldrich: a2074) were used at 1 : 200 dilution and 1 : 10000 dilution, respectively. Immunoblots were done through enhanced chemiluminescence detection (Amersham Biosciences: RPN2132). ECL blots were analyzed using a Fuji Film Intelligent Dark box (LAS-3000) and the associated computer programs (Image Reader Las-3000 and Multi-Gauge V2.3).

### 2.7. In Vitro Substrate-Independent Polyubiquitination

In vitro polyubiquitination assays were performed as described in Hagglund et al. [28], in ubiquitination buffer (50 mM Tris, pH 7.5, 2.5 mM MgCl_2_, 0.5 mM DTT) containing 40 ng of E1 (Calbiochem: 662070), 40 ng of one E2 (Biomol Affiniti, [His_6_]-UbcH1 (uw9020); [His_6_]-UbcH2 (uw9025); [His_6_]-UbcH3 (uw8730); [His_6_]-UbcH5a (uw9050); [His_6_]-UbcH5b (uw9060); [His_6_]-UbcH5c (uw9070); [His_6_]-UbcH6 (uw8710); [His_6_]-UbcH7 (uw9080); [His_6_]-UbcH10 (uw8715)) or [His_6_]-HR6A or [His_6_]-HR6B both produced in the laboratory, 2 *μ*g of biotinylated ubiquitin (Biomol Affiniti: uw8705), 0.2 mM ATP, 1 mM creatine phosphate (Calbiochem: 2380), and 15 units of porcine heart creatine phosphokinase (Calbiochem: 238395) in 30 *μ*L final volume. When indicated, the reaction was performed in absence of ATP. Immunoprecipitated RFP and either five *μ*g of GST or GST-RING/RFP recombinant protein were added to the reaction mixture and incubated at 37°C for 90 minutes. Reactions were stopped with 4X Laemmli buffer and subjected to SDS-Page analysis. Membranes were rinsed and blocked in PBS, 0.1% BSA, and 0.1% Tween 20 and reacted 1 hour at room temperature with 0.02 unit/mL of streptavidin peroxidase (Roche: 1089153). Biotinylated ubiquitin conjugates were revealed after ECL. Membranes were exposed on Kodak film (X-Omat LS film: ref F0899-50EA).

### 2.8. Immunofluorescence Analysis

Immunofluorescence labeling has been performed on testis frozen sections, on total germ cell preparations, or on elutriated germ cells prepared as previously described [26]. 

Cells were fixed in cold ethanol (−20°C) for 1 hour before a 30-minute blocking step in PBS containing 1% BSA (Sigma: A-4503) and 20% normal human serum. 

Colabeling experiments have all been performed in a sequential manner according to the same protocol. Antibodies were diluted in PBS containing 1% BSA and 2% normal human serum. After each antibody incubation, samples were washed three times with PBS containing 1% BSA. Primary specific antibodies were incubated overnight at 4°C and the secondary antibody was added for 1 hour at room temperature. The second specific antibody was then incubated for 2 hours at room temperature and the secondary antibody was added for 1 hour. After a final wash in PBS, slides were mounted in Vectashield reagent with DAPI (Vector Laboratories, Inc., Burlingame). 

The specific antibodies were: a goat polyclonal IgG Lamin B1 antibody (Santa Cruz Biotechnology: M-20, sc-6217); a rabbit polyclonal RFP (IBL Japan: JP 18791) antibody; a mouse monoclonal SUMO-I antibody (Zymed laboratories: 33-2400), a mouse IgG anti phosphohistone H2AX antibody (Stress gen Bioreagents corp., Canada: KAM-CC255) respectively diluted at 1 : 80, 1 : 150; 1 : 400 and 1 : 800.

The secondary antibodies (dilution 1 : 400) were: a Cy-3 conjugated affinity Pure donkey anti-goat IgG (Jackson Laboratories: 705-165-147); a goat anti-rabbit Alexa 516 and a goat anti-mouse Alexa 546 antibodies (Molecular Probes, Oregon: A11035 and A11030).

### 2.9. Image Analysis

Image acquisition (Objective 60 × 1.4 Uplanapo) and deconvolution using the constrained iterative algorithm (Softworx 2.5) was performed with Applied Precision DeltaVision system (Applied Precision, Issaquah, WA) built on an Olympus IX-70 base. 

In Figures 4(a) and 4(b) images were made by the sum of the intensities of three consecutive images taken in *Z* position (0.15 nm interval) in each channel. Treatment was performed using ImageJ NIH software. The 3D reconstruction (Figures 4(c), 4(d), and 4(e)) has been made with 30 consecutives images taken in *Z* position and the reconstruction is made using Volocity software and Image plugin Volume Viewer (Kai Uwe Barthel).

### 2.10. Immunogold Analysis

Samples fixation, ultrathin sections and immunogold labeling was performed as previously described [29]. Anti-RFP and anti-SUMO-I antibodies were diluted 1 : 100 in PBS; 1% BSA; 1% NGS. Colloidal gold conjugated anti-rabbit IgG antibodies (15 nm) and colloidal gold conjugated anti-mouse IgG antibodies (10 nm) were diluted 1/20 in PBS 0.01% fish-skin gelatin. Preparations were observed with a Philips CM12 electron microscope operating at 80 kV. No signal was observed when the first antibody was omitted.

## 3. Results

### 3.1. RFP Exhibits Various Localizations in Meiotic and Postmeiotic Germs Cells

**Figure 1:**
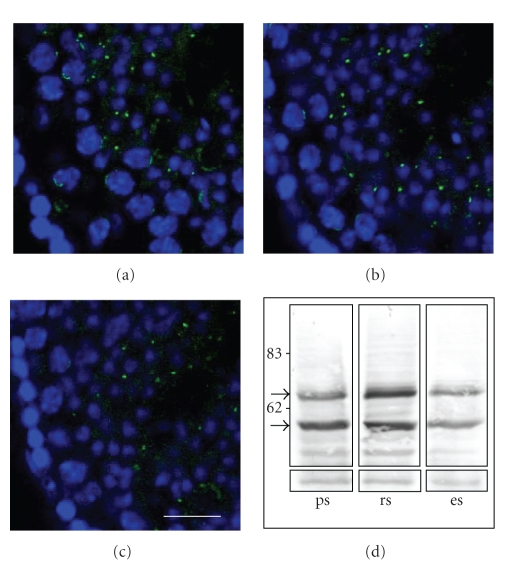
Distribution of RFP isoforms in meiotic and postmeiotic germ cells. (a)–(c) Immunofluorescent labeling of RFP on testis sections corresponding to late VIII and IX stages of mouse spermatogenesis. Sequential optical layers are separated by 4 *μ*m from each other. RFP positive signals (green) are detected either as a cap-like structure localized at the border of the nucleus (DAPI, blue) in pachytene spermatocyte (mainly seen in (a)) or as a dot in post meiotic germ cells and in residual bodies (mainly seen in (b) and (c)). Scale bar, 25 *μ*m. (d) 20 *μ*g of proteins prepared from 80%–90% pure elutriated preparations of pachytene spermatocytes (ps), round spermatids (rs), and elongated spermatids (es) were analyzed by western blotting with rabbit anti-RFP polyclonal antibody. Sizes (kDa) are indicated on the left side and the two RFP isoforms (58 and 68 kDa) are indicated by the arrows. A nonrelevant band was used as loading control (lower part).

RFP localization in mouse male germ cells, and in particular in pachytene spermatocytes, has previously been described as a perinuclear cap structure [9]. Immunolabeling of RFP in testis sections corresponding to stages late VIII and IX of mouse spermatogenesis (Figures 1(a)–1(c)) revealed at least two different localizations for RFP in germ cells: a thin cap-like structure forms next to the nucleus of meiotic germ cells which then seems to condense to a spot in later stages of spermatogenesis. Optical and electronic analysis indicate that these foci of RFP are then found in residual bodies (data not shown). 

Two isoforms of RFP 58 kDa and 68 kDa have previously been described in testis [9]. Tezel et al. [11] have shown that the 68 kDa form resulted from an O-glycosylation of RFP. Considering the specific localization of RFP protein in the nuclear matrix of pachytene spermatocyte, we checked the possible relationship between this posttranslational modification and the localization of RFP. We investigated the respective distribution of the 58 kDa and 68 kDa isoforms in elutriated germ cell populations (Figure 1(d)). Our results indicated that maximal RFP expression is found as expected in round spermatids [9]. However, both isoforms were found in pachytene spermatocytes, in round spermatids, and in elongated spermatids. Furthermore, the ratios between the two isoforms were equivalent in these different germ cell populations (53.43%/46.57% in pachytene spermatocyte, 51.68%/48.32% in round spermatids and 49.88%/50.12% in elongated spermatids for the ratio of 58 kDa/68 kDa isoform, resp.). Relative distribution of each RFP isoform does not show any clear relationship between the level of O-glycosylation and the localization of RFP in pachytene cells.

### 3.2. RFP Defines a Region Underlying the XY Body in Pachytene Spermatocyte

**Figure 2:**
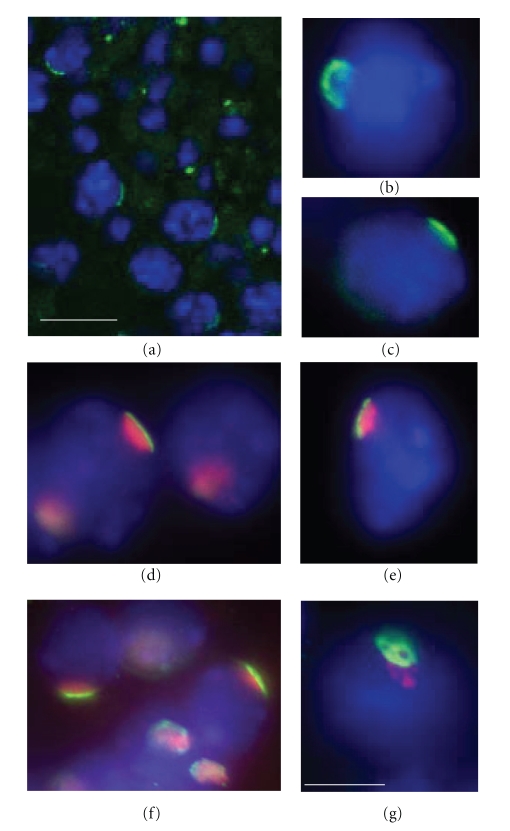
RFP defines a region underlying the XY body in pachytene spermatocyte. Immunofluorescent labeling of RFP on seminiferous tubule ((a), bar, 20 *μ*m) or in pachytene spermatocytes (b), (c). Coimmunolocalization of RFP and *γ*H2AX (d), (e) and RFP and SUMO-I (f), (g) in pachytene spermatocytes (bar, 8 *μ*m). In pachytene spermatocytes, RFP immunofluorescent staining (green) is condensed in a cap-like structure localized at the border of the nucleus (DAPI, blue). The XY body is visualized either by *γ*H2AX or SUMO-I antibodies (red). Superposition of labeling is never observed. The lace structure of RFP is evidenced when seen full face (g).

It has been reported that PIASy and SUMO-I localizes within the XY body, an intranuclear structure of the meiotic spermatocytes. Based on monolabeling immunostaining experiments, it has been proposed that RFP was located at the same place [7]. In order to examine the precise localization of endogenous RFP compared to XY body markers, such as SUMO-I and *γ*H2AX proteins [30, 31], we have performed coimmunolabeling experiments at the structural and the ultrastructural levels on meiotic spermatocytes. We demonstrate that RFP localized as a small cap at the periphery of the pachytene spermatocyte nuclei (Figure 2). Colabeling experiments made (Figures 2(d) and 2(e)) indicated that RFP and *γ*H2AX proteins did not colocalize but rather defined two different domains around and within the XY body. Specifically, *γ*H2AX is detected inside the XY body and RFP is a distinct layer adjacent to the XY body, on the side facing the nuclear membrane. These results were confirmed using anti-RFP and anti-SUMO-I antibodies giving a similar pattern (Figures 2(f) and 2(g)). To our knowledge, RFP is the only protein that has been shown to exhibit such a particular localization. 

**Figure 3:**
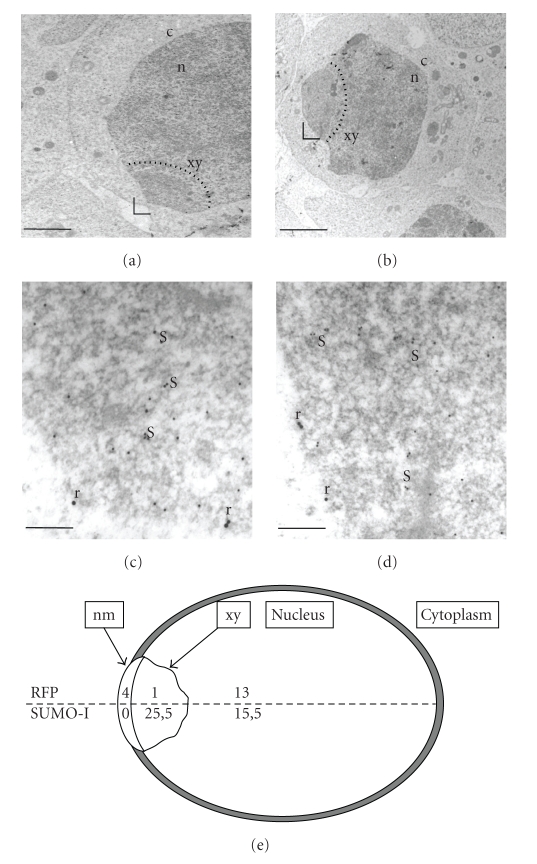
Subcellular localization of RFP and SUMO-I in pachytene spermatocyte nucleus. (a), (b) Overviews of pachytene cells in adult mouse testis (bar, 4 *μ*m). XY body (xy) in the nucleus (n) is underlined by the dotted line (c, cytoplasm). The indicated part of the XY body (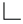) is enlarged in panels (c) and (d), respectively. (c), (d) RFP and SUMO-I coimmunolabeling detected using gold particles of two different sizes: 15 nm for RFP and 10 nm for SUMO-1 (bar, 160 nm). RFP (r) is significantly concentrated at the border of the nucleus adjacent to the XY body labeled by SUMO-I (s). (e) Nucleus, XY body, and nuclear membrane have been schematized respecting relative size (excepted for nuclear membrane). Mean of beads count is indicated in the corresponding area (nm, nuclear membrane facing the XY body, xy: XY body).

Electron microscopy analysis of immunogold labeling, performed on testis sections using anti-RFP and anti-SUMO-I antibodies, confirmed that the two proteins were very closely apposed but did not colocalize within spermatocyte nuclei (Figure 3). In order to obtain semiquantitative data, we have evaluated the number of 15 nm gold beads (corresponding to RFP) and the number of 10 nm gold beads (corresponding to SUMO) in Figure 3. To compare the localization of SUMO and RFP, we have evaluated the number of gold beads located in the nucleus and in the XY body. In Figure 3(a), nuclear surface has been evaluated to 59.48 *μ*m^2^ and the surface of the XY body to 2.68 *μ*m^2^. By counting the beads in the nucleus, we found a concentration of 0.25 beads/*μ*m^2^ for SUMO labeling and of 0.17 beads/*μ*m^2^ for RFP labeling. Within the XY body, we have obtained 9.32 beads/*μ*m^2^ for SUMO labeling and 0 beads/*μ*m^2^ for RFP labeling. Comparable results were obtained when Figure 3(b) was analyzed in the same way. Moreover, in Figure 3(a), the total length of nuclear envelope has been evaluated to 23.40 *μ*m and the membrane facing the XY body to 2.75 *μ*m. Three 15 nm gold beads have been found localized at the nuclear membrane, all of them facing the XY body. The same analysis was performed on Figure 3(b). Once again, 15 nm gold beads have been found only on the nuclear membrane facing the XY body. So, these observations confirm that RFP is localized at the border of the nucleus facing the XY body, while SUMO-I is inside the nucleus, associated with the XY body. These results were summarized in Figure 3(e).

### 3.3. RFP Takes Place of Lamin B1 in Region of the Nuclear Membrane Adjacent to the XY Body

**Figure 4:**
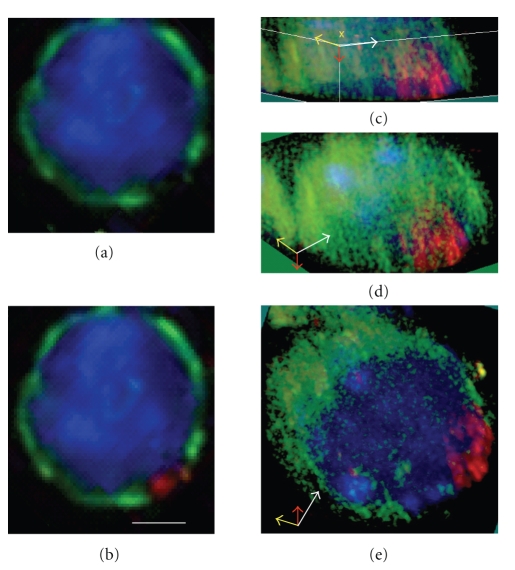
Relationship between XY body, RFP, and nuclear matrix in pachytene spermatocyte. Coimmunofluorescent labeling of lamin B1 (green) with RFP (red). (a), (b) At this cell stage, RFP takes place of lamin B1 along the border of the nucleus (DAPI, blue; bar, 8 *μ*m). (c)–(e) Data presented as volumes within a 3D-(xyz)-space reconstructed from an RGB image stack. The three dimensions are represented by oriented arrows (x: yellow, y: white, and z: red arrows), considering xyz reference positions taken in (c).

We have noticed that the labeling of the nuclear membrane of pachytene spermatocyte with an anti-lamin B1 antibody is interrupted in a region reminiscent of the size and the position of RFP and XY body [5]. Colabeling experiments using total germ cell preparation, anti-lamin B1 and anti-RFP antibodies showed that RFP accumulated in a region where lamin B1 was almost completely absent (Figures 4(a) and 4(b)). After deconvolution and 3D reconstruction, we visualized RFP labeling as a disc inside the nuclear matrix (Figures 4(c)–4(e)). When colabeling experiments were done with lamin B1 antibody and either SUMO-I or *γ*H2AX antibodies, we could confirm that this region of the nuclear envelope adjacent to the XY body was devoid of lamin B1 labeling (data not show). According to our results, we conclude that in the nuclear lamina of pachytene spermatocyte, the absence of lamin B1 could be involved in the specific localization of RFP adjacent to the XY body, and thus, RFP could provide a specific attachment point for sex chromosomes.

### 3.4. RFP Has an E3 Ubiquitin Ligase Activity Associated with Its RING Domain

**Figure 5:**
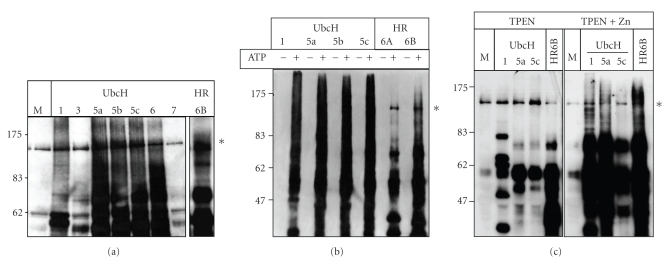
RFP has an E3 ubiquitin ligase activity. (a)–(c) Streptavidin peroxidase revelation of substrate-independent in vitro ubiquitination reactions using immunoprecipitated RFP (a) or GST-RING-B-box of RFP (GST-RING/RFP) (b), (c) as potential E3 ubiquitin ligase enzyme. Dark smears and signals higher than the biggest protein in the assay (monoubiquitinated E1 enzyme indicated by the asterisk) reflected polyubiquitinated products. E2 enzymes are indicated on the top part of each figure. M: master mix containing all the components of the system except E2 enzymes. Sizes (kDa) are indicated. (a) RFP protein immunoprecipitated from 15P-1 cell overexpressing Myc-RFP. Polyubiquitination is observed when UbcH1, UbcH5a, 5b, 5c, UbcH6, or HR6B are used as E2 enzymes. (b) Reactions are performed in presence or in absence of ATP. As expected mono- and poly-ubiquitinations are only observed in presence of ATP. (c) Prior to the substrate-independent in vitro ubiquitination reaction, GST-RING/RFP was pretreated with TPEN (left panel) in order to chelate Zn^2+^ and destroy RING finger architecture, or pretreated with TPEN, washed, and incubated with an excess of Zn^2+^ (right panel). As expected, when GST-RING/RFP was treated with TPEN, only mono-ubiquitination can be observed (left panel) and incubation of TPEN treated GST-RING/RFP with an excess of Zn^2+^ restores E3 ubiquitin ligase activity (right panel).

In somatic cells, RFP has been involved in transcriptional regulation. However, regarding its main localization in meiotic and postmeiotic germ cells, we looked for another function for RFP. RFP contains a RING domain and RING finger proteins have been proposed to be involved in degradation via the proteasome [28, 32, 33]. We first tested the ability of an Myc-tagged RFP to promote an in vitro substrate-independent polyubiquitination [28]. As shown in Figure 5(a), a dark smear, characteristic of polyubiquitination, can be observed with specific E2 enzymes such as UbcH1, UbcH5A, 5B, 5C, UbcH6, and HR6B. 

Using a GST-RING/RFP fusion protein, we demonstrate that the RING domain isolated from RFP contained the E3 ubiquitin ligase activity. This activity was strictly dependent of ATP hydrolysis (Figure 5(b)). The mandatory presence of Zn^2+^ was demonstrated by using TPEN, a chelator of divalent cations, which abolished the E3 activity (Figure 5(c), left panel). As expected, after readdition of an excess of Zn^2+^, the GST-RING/RFP retrieved its polyubiquitination activity (Figure 5(c), right panel). Control treatments with vehicle only or vehicle plus Zn^2+^ did not affect the E3 activity of the GST-RING/RFP protein (data not shown).

## 4. Discussion

We report here that in pachytene spermatocytes, RFP defines a precise and restricted region of nuclear matrix that correlates with the position of the XY body. This observation is supported by colabeling experiments using both deconvolution and electron microscopy. To our knowledge, the localization of RFP, associated with the nuclear matrix in a region juxtaposed to the XY body but not within, is unique in regard to the XY body. Of the lamins expressed in somatic cells, lamin B1 is the only one that could be detected in spermatocytes [5, 6]. Colabeling experiments using anti-lamin B1 and anti-RFP antibodies indicate that RFP occupied a region in the nuclear matrix, facing the XY body and devoided of lamin B1. Regarding the ability of RFP to interact with double-strand DNA and with nuclear matrix proteins [10], our data suggest that RFP could be involved in both positioning of the XY body in pachytene matrix spermatocyte and attachment of this structure to a region of the nuclear envelope matrix devoid of lamin B1. Whether this is mediated through strong association of RFP with other nuclear lamins or other proteins present in the XY body remains to be determined. However, it is unknown whether the localization of RFP is causative of XY body localization or is the result of the apposition of the XY body close to the nuclear membrane. Moreover, one cannot exclude that RFP removes lamin B1 from the region of the nuclear matrix where XY body attaches, possibly through its potential E3 ubiquitin ligase activity. All these hypotheses should be tested in future studies to better understand the underlying mechanisms. 

Neither O-glycosylation nor sumoylation of RFP seem to be associated with its specific localization in pachytene spermatocytes. Indeed, RFP (58 kDa) and O-glycosylated RFP (68 kDa) are evidenced at equivalent ratios in meiotic and postmeiotic germ cells (Figure 1(d)). Matsuura et al. [7] have reported that overexpression of SUMO-I in cell culture leads, in somatic cells, to the appearance of a 90 kDa sumoylated RFP. They also have proposed that RFP sumoylation could be involved in the localization of RFP in pachytene spermatocytes. However, we did not detect the described 90 kDa SUMO-I-RFP reactive band on western blot analysis performed on extracts enriched in pachytene spermatocytes (Figure 1(d)). Moreover, our results indicate that SUMO-I and RFP were closely located in spermatocytes but did not colocalize in the XY body (Figures 2 and 3). 

The results reported here showed that RFP is able to act as an E3 ubiquitin ligase with a series of E2s such as UbcH1, UbcH5A, 5B, 5C, UbcH6, and HR6B. This activity is exerted by the RING-B-Box domain and requires both ATP and Zn^2+^. HR6B has been described to be required for correct spermatogenesis and to be localized in the XY body [34, 35]. We propose that a functional interaction between RFP and HR6B could account for an essential role of RFP during spermatogenesis. These hypotheses have to be further investigated in vivo and yeast double-hybrid experiments are currently under way in our laboratory to test a possible interaction between RFP and HR6B. 

Like RFP, other E3 proteins such as RNF4, BRCA1, and ICP0 have also been initially described for their broad effect on the cellular transcription activity [28, 32, 33]. It is then interesting to note that, in somatic cells, these proteins, as well as RFP and polyubiquitinated products, colocalize with PML bodies and are able to self-assemble in such structures [12, 25, 36, 37]. We have demonstrated that RFP proteins assemble as a cap in the nuclear envelope matrix, facing the XY body. Later in spermatogenesis, RFP condenses as a spot in round spermatids and these foci are subsequently found in residual bodies, evocating a degradation process (Figure 1). One can postulate that these foci structures result from RFP self-assembly. These observations imply that RFP persists through spermatogenesis and is probably polyubiquitinated and degraded. We propose that RFP acts as an E3 ubiquitin ligase, in addition to its previously described role as transcriptional regulator in somatic cells [17, 18]. One attractive hypothesis could be that RFP, via its E3 ubiquitin ligase activity, might be involved in the release of XY body from the nuclear matrix when pachytene spermatocytes progress through meiotic prophase I. Whether the RFP containing foci structures are the cause or the consequence of XY body release remains to be determined. 

It is also interesting to consider the link between PML bodies and XY bodies. We know that both structures share common proteins, for example, PML, DAXX, BRCA1, SUMO-I, and *γ*H2AX, involved in polyubiquitination processes and are sites where transcriptional repression can be established and maintained [25, 31, 38]. So, some of these proteins are able to establish and maintain transcriptional repression by sequestrating transcriptional regulators far away from their targets [25, 38]. Therefore, one might postulate that, in meiotic cells, RFP could interact with transcriptional regulators and that these interactions could play a role in the transcriptional silencing of the XY body through RFP E3 activity.

## References

[1] Hoyer-Fender S (2003). Molecular aspects of XY body formation. *Cytogenetic and Genome Research*.

[2] Handel MA (2004). The XY body: a specialized meiotic chromatin domain. *Experimental Cell Research*.

[3] Scherthan H, Weich S, Schwegler H, Heyting C, Harle M, Cremer T (1996). Centromere and telomere movements during early meiotic prophase of mouse and man are associated with the onset of chromosome pairing. *Journal of Cell Biology*.

[4] Metzler-Guillemain C, Usson Y, Mignon C (2000). Organization of the X and Y chromosomes in human, chimpanzee and mouse pachytene nuclei using molecular cytogenetics and three-dimensional confocal analyses. *Chromosome Research*.

[5] Moss SB, Burnham BL, Bellve AR (1993). The differential expression of lamin epitopes during mouse spermatogenesis. *Molecular Reproduction and Development*.

[6] Alsheimer M, von Glasenapp E, Hock R, Benavente R (1999). Architecture of the nuclear periphery of rat pachytene spermatocytes: distribution of nuclear envelope proteins in relation to synaptonemal complex attachment sites. *Molecular Biology of the Cell*.

[7] Matsuura T, Shimono Y, Kawai K (2005). PIAS proteins are involved in the SUMO-1 modification, intracellular translocation and transcriptional repressive activity of RET finger protein. *Experimental Cell Research*.

[8] Takahashi M, Ritz J, Cooper GM (1985). Activation of a novel human transforming gene, ret, by DNA rearrangement. *Cell*.

[9] Tezel G, Nagasaka T, Iwahashi N (1999). Different nuclear/cytoplasmic distributions of RET finger protein in different cell types. *Pathology International*.

[10] Isomura T, Tamiya-Koizumi K, Suzuki M (1992). RFP is a DNA binding protein associated with the nuclear matrix. *Nucleic Acids Research*.

[11] Tezel G, Shimono Y, Murakumo Y (2002). Role for O-glycosylation of RFP in the interaction with enhancer of polycomb. *Biochemical and Biophysical Research Communications*.

[12] Cao T, Duprez E, Borden KLB, Freemont PS, Etkin LD (1998). Ret finger protein is a normal component of PML nuclear bodies and interacts directly with PML. *Journal of Cell Science*.

[13] Morris-Desbois C, Bochard V, Reynaud C, Jalinot P (1999). Interaction between the Ret finger protein and the Int-6 gene product and co-localisation into nuclear bodies. *Journal of Cell Science*.

[14] Shimono Y, Murakami H, Hasegawa Y, Takahashi M (2000). RET finger protein is a transcriptional repressor and interacts with enhancer of polycomb that has dual transcriptional functions. *Journal of Biological Chemistry*.

[15] Shimono Y, Murakami H, Kawai K, Wade PA, Shimokata K, Takahashi M (2003). Mi-2*β* associates with BRG1 and RET finger protein at the distinct regions with transcriptional activating and repressing abilities. *Journal of Biological Chemistry*.

[16] Shimono K, Shimono Y, Shimokata K, Ishiguro N, Takahashi M (2005). Microspherule protein 1, Mi-2*β*, and RET finger protein associate in the nucleolus and up-regulate ribosomal gene transcription. *Journal of Biological Chemistry*.

[17] Bloor AJC, Kotsopoulou E, Hayward P, Champion BR, Green AR (2005). RFP represses transcriptional activation by bHLH transcription factors. *Oncogene*.

[18] Krutzfeldt M, Ellis M, Weekes DB (2005). Selective ablation of retinoblastoma protein function by the RET finger protein. *Molecular Cell*.

[19] Cao T, Shannon M, Handel MA, Etkin LD (1996). Mouse Ret finger protein (rfp) proto-oncogene is expressed at specific stages of mouse spermatogenesis. *Developmental Genetics*.

[20] Meroni G, Diez-Roux G (2005). TRIM/RBCC, a novel class of ‘single protein RING finger’ E3 ubiquitin ligases. *BioEssays*.

[21] Reymond A, Meroni G, Fantozzi A (2001). The tripartite motif family identifies cell compartments. *The EMBO Journal*.

[22] Freemont PS (2000). Ubiquitination: RING for destruction?. *Current Biology*.

[23] Jackson PK, Eldridge AG, Freed E (2000). The lore of the RINGs: substrate recognition and catalysis by ubiquitin ligases. *Trends in Cell Biology*.

[24] Rassoulzadegan M, Paquis-Flucklinger V, Bertino B (1993). Transmeiotic differentiation of male germ cells in culture. *Cell*.

[25] Lopez P, Jacob RJ, Roizman B, Van Sant C (2002). Overexpression of promyelocytic leukemia protein precludes the dispersal of ND10 structures and has no effect on accumulation of infectious herpes simplex virus 1 or its proteins. *Journal of Virology*.

[26] Vidal F, Lopez P, Lopez-Fernandez LA (2001). Gene trap analysis of germ cell signaling to Sertoli cells: NGF-TrKA mediated induction of Fra1 and Fos by post-meiotic germ cells. *Journal of Cell Science*.

[27] Gu H, Roizman B (2003). The degradation of promyelocytic leukemia and Sp100 proteins by herpes simplex virus 1 is mediated by the ubiquitin-conjugating enzyme UbcH5a. *Proceedings of the National Academy of Sciences of the United States of America*.

[28] Hagglund R, Van Sant C, Lopez P, Roizman B (2002). Herpes simplex virus 1-infected cell protein O contains two E3 ubiquitin ligase sites specific for different E2 ubiquitin-conjugating enzymes. *Proceedings of the National Academy of Sciences of the United States of America*.

[29] Gillot I, Jehl-Pietr C, Gounon P (2005). Germ cells and fatty acids induce translocation of CD36 scavenger receptor to the plasma membrane of Sertoli cells. *Journal of Cell Science*.

[30] Fernandez-Capetillo O, Mahadevaiah SK, Celeste A (2003). H2AX is required for chromatin remodeling and inactivation of sex chromosomes in male mouse meiosis. *Developmental Cell*.

[31] Rogers RS, Inselman A, Handel MA, Matunis MJ (2004). SUMO modified proteins localize to the XY body of pachytene spermatocytes. *Chromosoma*.

[32] Ruffner H, Joazeiro CAP, Hemmati D, Hunter T, Verma IM (2001). Cancer-predisposing mutations within the RING domain of BRCA1: loss of ubiquitin protein ligase activity and protection from radiation hypersensitivity. *Proceedings of the National Academy of Sciences of the United States of America*.

[33] Häkli M, Lorick KL, Weissman AM, Jänne OA, Palvimo JJ (2004). Transcriptional coregulator SNURF (RNF4) possesses ubiquitin E3 ligase activity. *FEBS Letters*.

[34] Baarends WM, Wassenaar E, Hoogerbrugge JW (2003). Loss of HR6B ubiquitin-conjugating activity results in damaged synaptonemal complex structure and increased crossing-over frequency during the male meiotic prophase. *Molecular and Cellular Biology*.

[35] van der Laan R, Uringa E-J, Wassenaar E (2004). Ubiquitin ligase Rad18Sc localizes to the XY body and to other chromosomal regions that are unpaired and transcriptionally silenced during male meiotic prophase. *Journal of Cell Science*.

[36] Anton LC, Schubert U, Bacik I (1999). Intracellular localization of proteasomal degradation of a viral antigen. *Journal of Cell Biology*.

[37] Häkli M, Karvonen U, Janne OA, Palvimo JJ (2005). SUMO-1 promotes association of SNURF (RNF4) with PML nuclear bodies. *Experimental Cell Research*.

[38] Turner JMA, Aprelikova O, Xu X (2004). BRCA1, histone H2AX phosphorylation, and male meiotic sex chromosome inactivation. *Current Biology*.

